# Case report and literature review: Malignant adenomyoepithelioma after breast augmentation

**DOI:** 10.3389/fsurg.2022.981045

**Published:** 2022-10-13

**Authors:** Longqing Hu, Bei Qian, Zhecheng Yan, Kaijian Bing, Li Mei, Xincai Qu

**Affiliations:** ^1^ Department of Thyroid and Breast Surgery, Union Hospital, Tongji Medical College, Huazhong University of Science and Technology, Wuhan, China; ^2^ Department of Pathology, Union Hospital, Tongji Medical College, Huazhong University of Science and Technology, Wuhan, China

**Keywords:** breast augmentation, malignant adenomyoepithelioma, MAME, prosthetic implantation, oncological safety, breast reconstruction

## Abstract

**Background:**

Breast malignant adenomyoepithelioma (MAME) after breast augmentation has never been reported.

**Case summary:**

We reported a case of a 55-year-old woman who was diagnosed with breast MAME 16 years after breast augmentation. Breast augmentation was performed on the patient with two 200 ml round textured prostheses in the subpectoral plane through axillary incisions in 2004. However, a breast ultrasound in 2020 revealed a suspicious malignant lump in the right breast, which was finally confirmed as MAME by pathology. Skin-sparing modified radical mastectomy and immediate breast reconstruction with expander implantation were performed. Subsequently, the patient received three cycles of chemotherapy with the regimen of anthracycline and cyclophosphamide. In the following nearly 2 years of follow-up, no tumor recurrence and metastasis were found, and the overall treatment was satisfactory for the patient.

**Conclusion:**

Here, we present a unique case in which a patient was diagnosed with breast MAME after breast augmentation. Skin-sparing modified radical mastectomy and immediate breast reconstruction with expander implantation are feasible approaches that yield at least short-term oncological safety and acceptable aesthetic results. However, whether there is a potential relationship between MAME and breast implants remains to be further explored. Meanwhile, due to the rarity of breast MAME, more authoritative strategies considering both oncological safety and aesthetics to seek better long-term therapeutic effects are needed.

## Background

Breast augmentation is the most commonly performed aesthetic surgical procedure. Silicone breast implants are used in nearly 300,000 breast augmentation and 100,000 breast reconstruction operations annually in the United States (1). Although several epidemiologic pieces of evidence show no link between implants and the risk of developing breast cancer (2), the research on breast cancer after augmentation mammoplasty is still attracting much attention. Early research suggested that cosmetic breast implants adversely affect breast cancer-specific survival following the diagnosis of such disease (3). Some authors have reported that breast cancers in augmented women present at a later stage are more aggressive tumors than those arising in nonaugmented women (4). It has also been reported that women with submuscular implants have a higher incidence of breast cancer than those with subglandular implants (5). In addition, there have been reports that women with textured breast implants are more likely to be diagnosed with anaplastic large cell lymphoma (ALCL) (6). All these studies remind a potential relationship between breast implants and breast tumors.

Adenomyoepithelioma (AME) is a rare tumor that can be seen in salivary glands, skin appendages, lungs, and breasts. Among them, AME of the breast was first reported by Hamperl in 1970 (7), which was considered to be a benign tumor formed by the biphasic proliferation of epithelial and myoepithelial cells. In the classification of breast tumors published by the World Health Organization in 2019, this kind of disease is clearly defined as breast epithelial–myoepithelial lesions. Two types of lesions composed of epithelial and myoepithelial cells in mammary ducts and/or tubules are seen under a microscope. Breast malignant adenomyoepithelioma (MAME) is a rare double-cell group lesion of mammary epithelial cells and myoepithelial cells, which means that one or both of them have malignant characteristics. So far, breast MAME after breast augmentation with implants has never been reported.

## Case description

### History of illness and physical examination

A 55-year-old female patient presented with a right breast lump by palpation without pain and nipple discharge. A medical history confirmed that the patient was implanted with two 200 ml round textured prostheses in the subpectoral plane through axillary incisions in 2004. There were no obvious adverse reactions such as infection and seroma after the breast augmentation surgery. No breast lump was detected before breast augmentation, and the family history of breast cancer was denied.

### Physical examination

Physical examination showed that bilateral breasts were symmetrical, with no nipple deviation and depression, no nipple bleeding or discharge, and no orange peel sign or dimple sign. In addition, the prosthesis could be reached in both breasts. A lump of about 3 cm could be reached under the right nipple with hard texture, unclear boundary, low mobility, and no obvious adhesion to the skin. There was no obvious mass in the left breast or enlarged lymph nodes in the bilateral axilla.

### Imaging examination

**Figure 1:**
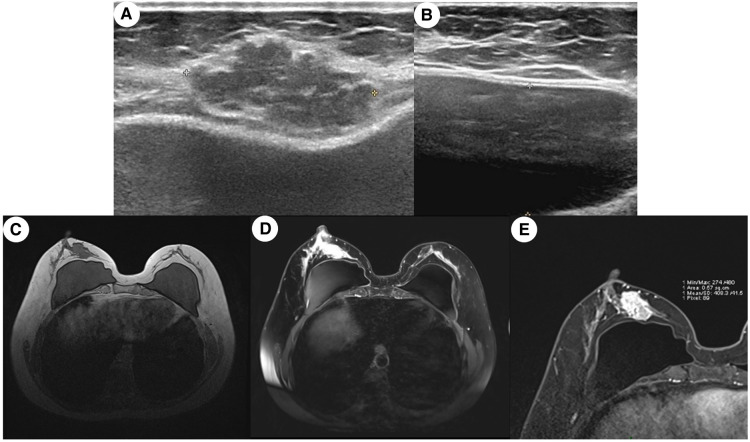
Medical imaging presentation of the breast mass. (**A**) Ultrasound image of a mixed-density multilobulated mass subcutaneous to the breast of an irregular shape and uneven margin; (**B**) ultrasound image of the pocket of breast prosthesis; (**C**) MRI image of the breast showing a lobulated mass in the right inner quadrant was adjacent to the margin of the prosthesis, and the vessels were increased and thickened; (**D**) contrast-enhanced MRI imaging showing that several early enhanced small nodules were seen in the upper posterior area of the tumor and the diffusion was limited, which seems like the satellite lesion of the tumor; (**E**) enlargement of the image in (**D)**. The capsule of the prosthesis was wrinkled, and the edge of the prosthesis presented the change of chronic inflammation.

The breast ultrasound on August 21, 2020 (Figures 1A,B), showed a lobulated and spiculated hypoechoic solid neoplasm of 28.8*24.5*13.9 mm in the inner lower quadrant of the right breast adjacent to the nipple. The neoplasm was measured about 4.8 mm away from the body surface. According to the Breast Imaging Reporting and Data System (BI-RADS), the tumor was classified as grade 5, which meant a malignant possibility. In addition, no obvious abnormality was found in bilateral axillary lymph and the bilateral prosthesis capsule was intact.

The MRI on August 24, 2020 (Figures 1C–E), confirmed the existence of phyllodes lump and thickening and increase of blood vessels, suspected as a malignant tumor with a grade of 4C of BI-RADS. At the same time, several early enhanced small nodules, which were suspected as the satellite lesions of the tumor, were found in the superior external area of the lump. Moreover, chronic inflammation-like changes were reported in the surrounding tissues of the prosthesis under the pectoralis major.

### Treatment and final diagnosis

**Figure 2:**
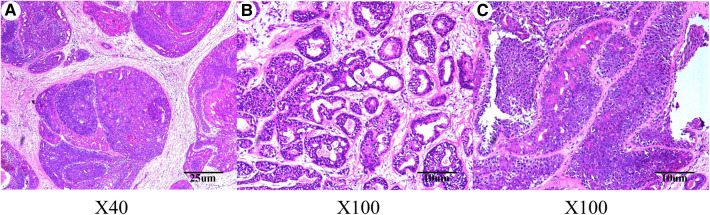
Hematoxylin–eosin staining indicated malignant adenomyoepithelioma of the breast lump. (**A**) Magnification of the main body of the lump (original magnification ×40); (**B**) magnification of the main body of the lump (original magnification ×100); (**C**) magnification of the different areas of the lump body (original magnification ×100).

**Figure 3:**
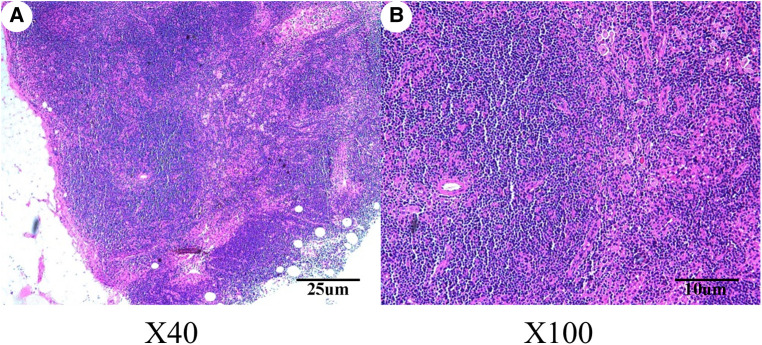
Hematoxylin–eosin staining revealed that no metastasis was observed in the sentinel lymph nodes of the right breast. (**A**) Magnification of the main body of the lymph nodes (original magnification ×40); (**B**) magnification of the main body of the lymph nodes (original magnification ×100).

On September 2, 2020, considering the patients’ aesthetic requirements, we attempted to remove the lump completely with a negative incisal margin. Unfortunately, the intraoperative pathological examination indicated the breast MAME and posterior margin of the nipple showed cancer involvement. Therefore, we had to excise the nipple–areola complex and perform the skin-sparing modified radical mastectomy. Although there was no established uniform recommendation for the time interval of replacing implants, most plastic surgeons recommend routine replacement no more than 15 years after initial placement (8). At the same time, considering the possibility of subsequent radiotherapy and chemotherapy for breast cancer, the prosthesis was removed, and immediate breast reconstruction with expander implantation was performed. As intraoperative pathological examination demonstrated that no cancer metastasis was found in four sentinel lymph nodes, therefore, the patient did not undergo axillary lymph node dissection. The pathological results are shown in Figures 2, 3. Immunohistochemistry shows the following tumor cells: ER (−), PR (+, about 5%, weak to moderate intensity), HER2 (1+), GATA-3 (+), AR (+, 40%, moderate intensity), E-cadherin (+), EMA (+), GCDFP-15 (−), CD-117 (focal +), Syn (−), SOX10, CK5/6, p63, S-100 and SMM-HC (myoepithelial +), and Ki67 (Li: about 30%). Because of a bidirectional differentiation of glandular and myoepithelium, it was diagnosed as AME; at the same time, due to obvious cell atypia, pathological mitosis, infiltration at the tumor edge, etc., it was diagnosed as MAME.

### Outcomes and follow-up

The patient recovered well after the operation with no complications and was discharged on September 10, 2020. Since November 11, 2020, she has received three cycles of chemotherapy in another hospital with the AC*4 regimen (anthracycline combined with cyclophosphamide) and then gave up on her own. There was no recurrence or metastasis in nearly 2 years of follow-up after the operation. This study was reported in agreement with the principles of the CARE guidelines (9).

## Discussion

The potential carcinogenicity of breast implants has always been a common concern for both doctors and patients. Although extensive data negate any link between the implants and an increased incidence of breast cancer (1, 10), a more rigorous recognition is that the evidence remains inconclusive about any association between silicone gel implants and long-term health outcomes (10). Moreover, the largest study of breast implants based on the long-term results of 9,9993 patients explicitly claimed that silicone implants are associated with an increased risk of certain rare harm (11). Meanwhile, Pan et al. suggested that the incidence of breast cancer for women with submuscular implants was higher than that for those with subglandular implants (5). All these views seem to support the presentation of this study to some extent. In addition, some scholars believe that cosmetic breast implants adversely affect breast cancer-specific survival following the diagnosis of such disease (3). However, some studies found that patients with implants were more likely to develop a cancer diagnosis compared with the general population. However, the data do not support breast implants being responsible for these findings. At the same time, some research studies indicated that women with breast implants have different patient demographics and lifestyles from the general population, which may also explain the finding. As for ALCL, reports from the scientific community have suggested a possible link between the disease and breast implants (6). Therefore, this study concludes that more authoritative, large-scale studies are needed to carefully elucidate the relationship between breast implants and long-term health outcomes. Also, whether there is a potential relationship between MAME and breast implants remains to be further explored.

Breast AME is a very rare breast tumor. Most of the relevant literature works are in the form of case reports. According to the statistics of AME cases reported in the literature since 2010, the age of onset of breast AME ranged from 27 to 83 years, with an average of about 60 years. Most of the cases were found in females, although male breast AME cases were also reported. The vast majority of breast AME cases are benign, and the most common site is the external superior quadrant of the breast; the longest course of the disease is 14 years, and the shortest is 4 days. The metastatic sites of AME include lymph nodes, lungs, brain, bone, thyroid, liver, kidneys, thoracic wall, soft tissue, and axillary lymph nodes (12). In the cases with metastasis, the number of distant metastases was significantly more than that of axillary lymph node metastases, so we believe that hematogenous metastasis is more common than lymph node metastasis in breast AME.

Breast AME is difficult to diagnose because of its low incidence rate. According to the clinical data, the most common clinical manifestation was a painless breast lump. Physical examination showed that the lump was medium or hard; most had no nipple discharge, but there were also records of bloody discharge. The most common ultrasound result was round or oval hypoechoic or mixed echo solid lobulated nodules with clear boundaries. Most of the breast molybdenum showed lobulated, irregular, fuzzy boundary, isodense, or slightly high-density lumps, and some of them had small central flake calcification. The clinical manifestations and ultrasound, mammography, and MRI findings of this disease are difficult to distinguish from other breast tumors, among which the most easily misdiagnosed is breast fibroadenoma. Therefore, the main method of diagnosis is surgical resection and pathological examination: pathological diagnosis includes morphological, biological, and immunohistochemical examination. Breast AME is easy to be misdiagnosed clinically, and it should be carefully differentiated from breast fibroadenoma, benign myoepithelioma, pleomorphic adenoma, adenoid cystic carcinoma, myoepithelial carcinoma, etc.

Meanwhile, it is difficult to distinguish between benign and malignant breast AMEs. The malignant features reported in the literature that may lead to recurrence and metastasis included cellular pleomorphism, increased mitotic activity, nuclear pleomorphism, prominent nucleoli, hyperchromasia, and necrosis. The above indexes should be considered comprehensively; otherwise, it is easy to be misdiagnosed. For MAME, the biological behavior seems to be related to the degree of malignant component and tumor size (13). The two components of AME (mammary epithelium and myoepithelium) can be malignant transformation and distant metastasis. Most of the time, it is the malignant transformation of only one of the components, but cases of two components malignant transformation at the same time have also been reported (14). Immunohistochemistry showed that the tumor was bipolar; in most cases, SMA, actin, p63, CK5/6, and S-100 were positive in myoepithelial cells, while ER/PR was negative. Some think that the combination of p63 and actin/troponin immunostaining is the most suitable method for visualizing myoepithelial cells (15). Most cases are often triple negative (ER/PR/HER-2 negative) (16), so endocrine therapy and anti-Her-2 therapy are often ineffective, which also leads to the difficulty of treatment. Tavassoli (17) divided breast AMEs into three types: (1) spindle cell type, in which the lesions were mainly composed of proliferative spindle myoepithelial cells mixed with a small number of epithelial cells; (2) tubular type, in which the lesions are composed of myoepithelial cells and glandular epithelial cells around the duct, similar to sclerosing papilloma, tubular adenoma, and glandular tubular adenoma; and (3) tubular type, in which the proliferative myoepithelial cells are arranged in a solid, nest-like arrangement, and some of them are like plasma cells, the cytoplasm is dense, transparent, eosinophilic, and the nucleus moves around. Additionally, recently, breast AMEs were characterized as PIK3CA, AKT1, and HRAS mutations (18).

At present, the treatment for breast AME has not been unified, but there are literature (19) records that breast AME has the risk of recurrence and metastasis. Therefore, for benign breast AME, we recommended local lump resection and keeping a safe incisal margin, while for malignant breast AME, we recommended modified radical mastectomy to ensure a negative margin; sentinel lymph node biopsy was used to decide whether axillary lymph node dissection is necessary. There is no clear data to support the effectiveness of chemotherapy, radiation therapy, and endocrine therapy. Whether patients with MAME should receive chemotherapy and radiotherapy is still controversial. We believe that it should be considered comprehensively from the tumor’s biological behavior and morphological behavior, invasion degree of surrounding tissues, lymph node metastasis, and patients’ will. Two cases of chemotherapy and one case of radiotherapy were recorded: one received TC (paclitaxel liposome and cyclophosphamide) regimens with four cycles; one received one cycle of TE regimen (docetaxel and epirubicin) before operation and another cycle of TE regimen after operation; one received radiotherapy [gray (Gy) 50 total dose plus a boost of Gy 10 to the tumor bed] (20). For metastatic breast AME, there is no definite reported treatment method, and its prognosis is very poor (21). No literature proves that radiotherapy and chemotherapy have therapeutic effects on metastatic breast AME, but some scholars (22) believe that eribulin may be beneficial to patients with metastatic breast AME.

Since there is no literature on MAME after augmentation mammoplasty, this first reported study may provide experience for managing such patients. Some limitations were present in this study. First, more details of augmentation mammoplasty were unknown; Second, the follow-up time was too short to assess the long-term outcomes. Third, we are unable to present preoperative and postoperative surgical photos of the patient due to the patient’s concern about privacy protection.

## Conclusion

Here, we present a unique case of a patient diagnosed with breast MAME after breast augmentation. Skin-sparing modified radical mastectomy and immediate breast reconstruction with expander implantation is a feasible approach that yields at least short-term oncological safety and acceptable aesthetic results. However, whether there is a potential relationship between MAME and breast implants remains to be further explored. Meanwhile, due to the rarity of breast MAME, more authoritative strategies considering both oncological safety and aesthetics to seek better long-term therapeutic effects are needed.

## Data Availability

The original contributions presented in the study are included in the article/Supplementary Material, further inquiries can be directed to the corresponding author.
